# Molecular and physical technologies for monitoring fluid and electrolyte imbalance: A focus on cancer population

**DOI:** 10.1002/ctm2.461

**Published:** 2021-06-20

**Authors:** Devasier Bennet, Yasaman Khorsandian, Jody Pelusi, Amy Mirabella, Patrick Pirrotte, Frederic Zenhausern

**Affiliations:** ^1^ Center for Applied NanoBioscience and Medicine The University of Arizona College of Medicine Phoenix USA; ^2^ HonorHealth Research Institute Scottsdale USA; ^3^ Collaborative Center for Translational Mass Spectrometry Translational Genomics Research Institute Phoenix USA

**Keywords:** biomarkers, biomedical sensors, electrolyte imbalance, health performance, hyperkalemia, hypocalcemia, hyponatremia, proteomics and metabolomics, wearables

## Abstract

Several clinical examinations have shown the essential impact of monitoring (de)hydration (fluid and electrolyte imbalance) in cancer patients. There are multiple risk factors associated with (de)hydration, including aging, excessive or lack of fluid consumption in sports, alcohol consumption, hot weather, diabetes insipidus, vomiting, diarrhea, cancer, radiation, chemotherapy, and use of diuretics. Fluid and electrolyte imbalance mainly involves alterations in the levels of sodium, potassium, calcium, and magnesium in extracellular fluids. Hyponatremia is a common condition among individuals with cancer (62% of cases), along with hypokalemia (40%), hypophosphatemia (32%), hypomagnesemia (17%), hypocalcemia (12%), and hypernatremia (1‐5%). Lack of hydration and monitoring of hydration status can lead to severe complications, such as nausea/vomiting, diarrhea, fatigue, seizures, cell swelling or shrinking, kidney failure, shock, coma, and even death. This article aims to review the current (de)hydration (fluid and electrolyte imbalance) monitoring technologies focusing on cancer. First, we discuss the physiological and pathophysiological implications of fluid and electrolyte imbalance in cancer patients. Second, we explore the different molecular and physical monitoring methods used to measure fluid and electrolyte imbalance and the measurement challenges in diverse populations. Hydration status is assessed in various indices; plasma, sweat, tear, saliva, urine, body mass, interstitial fluid, and skin‐integration techniques have been extensively investigated. No unified (de)hydration (fluid and electrolyte imbalance) monitoring technology exists for different populations (including sports, elderly, children, and cancer). Establishing novel methods and technologies to facilitate and unify measurements of hydration status represents an excellent opportunity to develop impactful new approaches for patient care.

## Graphical abstract

1. Electrolyte imbalance is a common complication in cancer that mainly involves sodium, potassium, and calcium alterations.
2. If the individual at risk is not monitored and hydrated, it leads to severe complications.
3. Hydration status assessed in various indices; plasma, sweat, saliva, urine, interstitial fluid, and skin interface have been widely used, but no unified (de)hydration monitoring technologies exist for different populations.

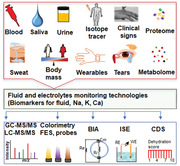

**Abbreviations**
AC: alternating current
ANG‐2: Asante NaTRIUM Green‐2
AVP: arginine vasopressin
BIA: bioelectrical impedance analysis
BIVA: bioelectrical impedance vector analysis
C: capacitance
CDS: clinical dehydration scale
CM: cell membranes
ECF: extracellular fluid
FES: flame emission spectrophotometry
GC: gas chromatography
HPLC: high‐performance liquid chromatography.
ICF: intracellular fluid
ISEs: ion selective electrodes
ISF: interstitial fluid
MS: mass spectrometry
PA: phase angle
PTH: parathyroid hormone
R: resistance
RANKL: receptor activator of nuclear factor‐κB ligand
Rc: reactance
RE: reference electrode
SBFI: Na^+^‐binding benzofuran isophthalate
osm: osmolality
SIADH: syndrome of inappropriate antidiuretic hormone
SiHG: silicone‐hydrogel contact lenses
TBF: total body fluid
WE: working electrode

## 1 INTRODUCTION

Dehydration is an excessive loss of water, often accompanied by electrolyte imbalance. Fluid and electrolyte imbalance is a significant clinical problem that is directly related to morbidity and mortality.^1^ Many factors can cause an imbalance between the electrolyte and water levels at all stages of life^2^ including aging, excessive or lack of fluid consumption, alcohol consumption, hot environment, diabetes, vomiting, diarrhea, cancer, radiation, chemotherapy, and use of diuretics. The issues central to fluid and electrolyte imbalances are of the utmost importance in cancer patients, and staying hydrated during cancer treatment is very important.^3^ Several clinical examinations showed an essential impact of electrolyte imbalance in cancer patients. Garces et al.^4^ studied 1088 consecutive electrolyte imbalance cases in oncology phase I clinical trials between 2011 and 2015 at the Drug Development Unit of the Royal Marsden Hospital. They showed elevated rates of hyponatremia (62%), hypokalemia (40%), hypophosphatemia (32%), hypomagnesemia (17%), and hypocalcemia (12%) in cancer patients treated with new anticancer agents. The report concluded that 8.46% of patients who developed dose‐limiting adverse events in terms of electrolyte imbalance during follow‐up had poor survival (overall survival for 26 weeks vs. 37 weeks is 95% CI: 1.37‐1.90; *P* < .001). In recent studies,^5^, ^6^, ^7^ patients who receive parenteral hydration in advanced cancer, improved comfort, dignity, and quality of life (overall median survival was increased, 95% CI, 13 to 21 days), suggest the importance of monitoring and adjusting electrolyte disorders in cancer patients. Currently, no real‐time standard monitoring system exists for evaluating dehydration in individuals with cancer. To maintain hydroelectrolytes homeostasis, water loss from the kidney, lungs, and skin must be mitigated by an appropriate oral or parenteral fluid and electrolytes. The disparity between end‐of‐life care in hospitals and hospice care also challenges the maintenance of fluid and electrolyte levels in cancer patients. Patients undergoing chemotherapies are also at high risk for dehydration and related complications due to multiple causes.^8^ Early diagnosis in these patients is difficult because thirst and physical signs of dehydration are often absent or misleading. If not prevented or treated, dehydration results in longer parenteral hydration and chemotherapy stay. Many hydration assessment methods currently exist for different purposes: clinical, sports, academic, military, or industrial areas. Early efforts in this area focused on physical symptoms that monitored mobility and vital signs such as body mass, intake and output measurements, stool number, temperature, heart rate, respiratory rate, skin turgor, thirst, and mucous membrane moisture. Additionally, many laboratory testing methods have been used to determine the hydration status, such as urine parameters (volume, color, protein content, specific gravity, and osmolality [osm]), blood parameters (hemoglobin concentration, hematocrit, plasma osmolality, plasma volume, and electrolytes concentration, plasma testosterone, adrenaline, noradrenaline, cortisol, and atrial natriuretic peptide), and pulse rate.^9^

**FIGURE 1:**
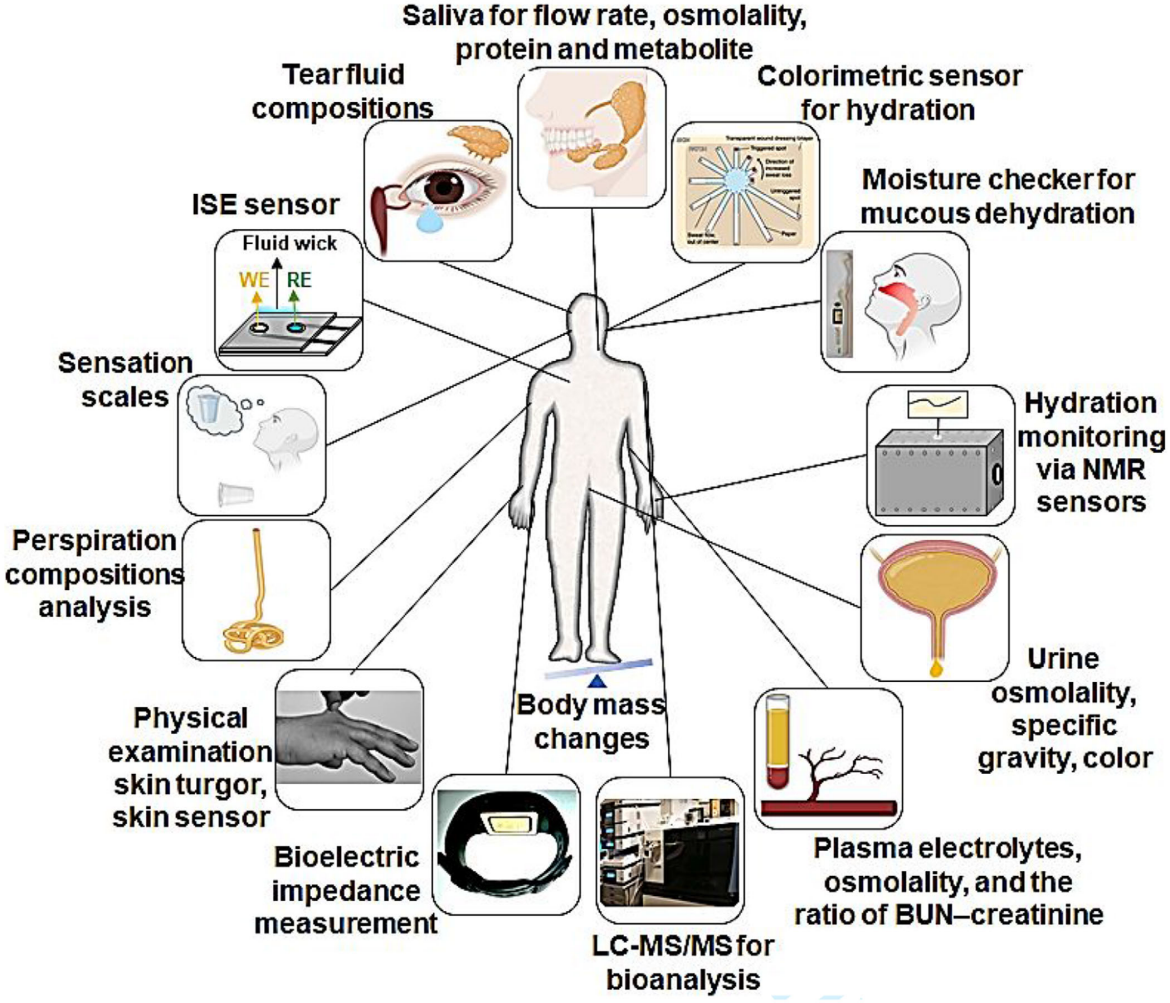
Representative examples of hydration monitoring techniques. Clockwise from top: Salivary biomarkers. Saliva parameters are the potential biomarker of hydration status.^23^ Skin patch for measuring sweat rate and hydration status (adapted from Ref. [24]). Mucosal dryness in dehydrated patients using an oral moisture‐checking device (Mucus®, Life Co. Ltd.) (adapted from Ref. [25]). Fluid assessment in patients by point‐of‐care NMR relaxometry (schematic representation from Ref. [26]). Water loss (intracellular dehydration) is assessed using urinary tests.^27^ Measurement of blood plasma parameters during progressive dehydration.^28^ Dehydration–rehydration biomarker discovery by LC‐MS/MS.^29^ BIA to estimate body fluid compartments.^10^ Assessment of hydration status by physical examination.^30^ Sweat analysis for electrolyte concentration on body water.^31^ Thirst sensation as a measure of hydration status.^32^ Ion‐selective electrodes (ISE) sensor for continuous electrolytes monitoring (concept adapted from Ref. [33]). Tear fluid parameters are the potential indices of hydration status.^34^ Body mass changes are indices of hydration status.^35^

Recently, monitoring (de)hydration status using wearable devices has increased in rehabilitation, sport, military, and performance‐related activities. The commercial hydration status monitoring systems mainly focus on noninvasive technologies, as illustrated in Table 1, which do not fully meet the requirement of fluid and electrolyte imbalance monitoring. Further research needs to be completed to confirm whether dehydration is associated with fluid loss or electrolyte imbalance. Researchers branch out from tracking hydration activity to focusing on tackling significant challenges in measuring electrolytes and their relation to hydration status. Figure 1 describes different diagnostic approaches to (de)hydration assessment. In order to understand the hydroelectrolytic imbalance and their pathophysiological responses, plasma,^18^ sweat,^19^ tears,^20^ saliva,^21^ urine,^21^ and interstitial fluid (ISF)^22^ have been used for hydration status monitoring. This review evaluates existing literature to determine the role and impact of dehydration in cancer therapy outcomes. It will also briefly discuss the pathophysiological implications of fluid and electrolyte imbalance in a cancer population and the different approaches that have been explored for assessing dehydration with a focus on those likely to be usable in the cancer population. Different approaches to identify novel hydration biomarkers in various populations using biochemical, electrochemical, proteomics, metabolomics, microneedle‐based transdermal sensor, bioelectrical impedance analysis (BIA), and wearable sensors will also be discussed.

**TABLE 1:** Selected examples of commercial hydration status monitoring systems

Product, company	Analyte, sample	Platform	Monitoring mechanism	Availability	Website	Key studies
GoBe2, Healbe Corp	TBW	Wrist‐band	Bioimpedance	Yes	https://healbe.com	Dehydration and rehydration^10^
Wear‐and‐forget, GE Research	Sweat electrolyte	Wearable patch	Ion‐selective electrodes (ISEs) with flexible microfluidics	UD	https://www.ge.com/research/project/wearable‐hydration‐status‐monitoring	Sweat rate and composition hydration status^11^
Hydra‐Alert Jr, Acumen	Fluid loss	Watch	Heat, humidity, heart rate	Yes	Acumen, Inc.	Dehydration during physical activity^12^
LVL Wearable, kickstarter	Skin hydration	Wrist‐band	Intensities by near‐infrared light	Yes	https://www.kickstarter.com	Degree of hydration in human skin^13^
Aura Band, Aura	Body composition	Wrist‐watch	Bioelectrical impedance vector	Yes	https://auraband.io	Body fluid volume^14^
B60, Nobo	Fluid balance monitor	Watch	Near‐IR to measure water	Yes	https://www.nobo.io	Dehydration and overhydration^15^
Halo H1, Halo	Interstitial fluid level, Na^+^‐K^+^	Smart watch	Optical and electrical sensing	UD	https://www.halowearables.com	Hydration levels in athletes^16^
ECHO Smart Patch, Kenzen	Sweat hydration, sodium	Peel‐and‐stick Patch	Epidermal sensor	UD	https://www.kenzen.com	Hydration levels in athletes^17^

Abbreviation: UD, underdevelopment.

## 2 PATHOPHYSIOLOGY OF ELECTROLYTES

### 2.1 Significance of the electrolytes

The human body consists of up to 24 elements, of which sodium (Na^+^), calcium (Ca^2+^), and potassium (K^+^) are the most common minerals^36^ with critical roles in electrolyte homeostasis. Electrolyte concentrations in various bodily fluids are shown in Table 2.^36^, ^37^ Having the right concentrations of these electrolytes is important for maintaining fluid balance among the intracellular fluid (ICF) and extracellular fluid (ECF) compartments. Of these electrolytes, Na^+^ is the principal cation and osmotic mediator of ECF, and K^+^ is in the ICF cation.^38^ Kidneys regulate ECF Na^+^ concentration.^39^ A total of 44% of the Na^+^ is in ECF, 9% in the ICF, and the rest 47% is in bone; from that, 50% are exchangeable.^38^ The ICF contains 98% of K^+,^ and the majority (75%) is stored in skeletal muscle.^39^ The lung‐renal functions allow regulating the levels of these ions in the ECF. The primary physiological role of Na^+^ is to control the EC volume and K^+^ via Na⁺/K⁺‐ATPase pump. The Na^+^/K^+^‐ATPase pumps 3 Na^+^ to EC and 2 K^+^ to IC for every single ATP depleted and helps to maintain osmotic equilibrium.^40^ The K^+^ homeostasis is maintained by daily intake (recommended at 4700 mg),^41^ active and passive renal excretion, and balance between the ICF and ECF. Potassium is exchanged with Na^+^ in the renal tubules through basolateral Na^+^/K^+^ pumps with the help of aldosterone by stimulating the activity of the Na^+^/K^+^‐ATPase. Ca^2+^ metabolism and homeostasis require a steady interaction between bone and ECF with the influence of several hormones. Ca^2+^ is absorbed from the intestines under activated vitamin D, which plays a crucial role in blood Ca^2+^ homeostasis.^42^

### 2.2 Pathophysiology of electrolyte imbalance in cancer patients

**FIGURE 2:**
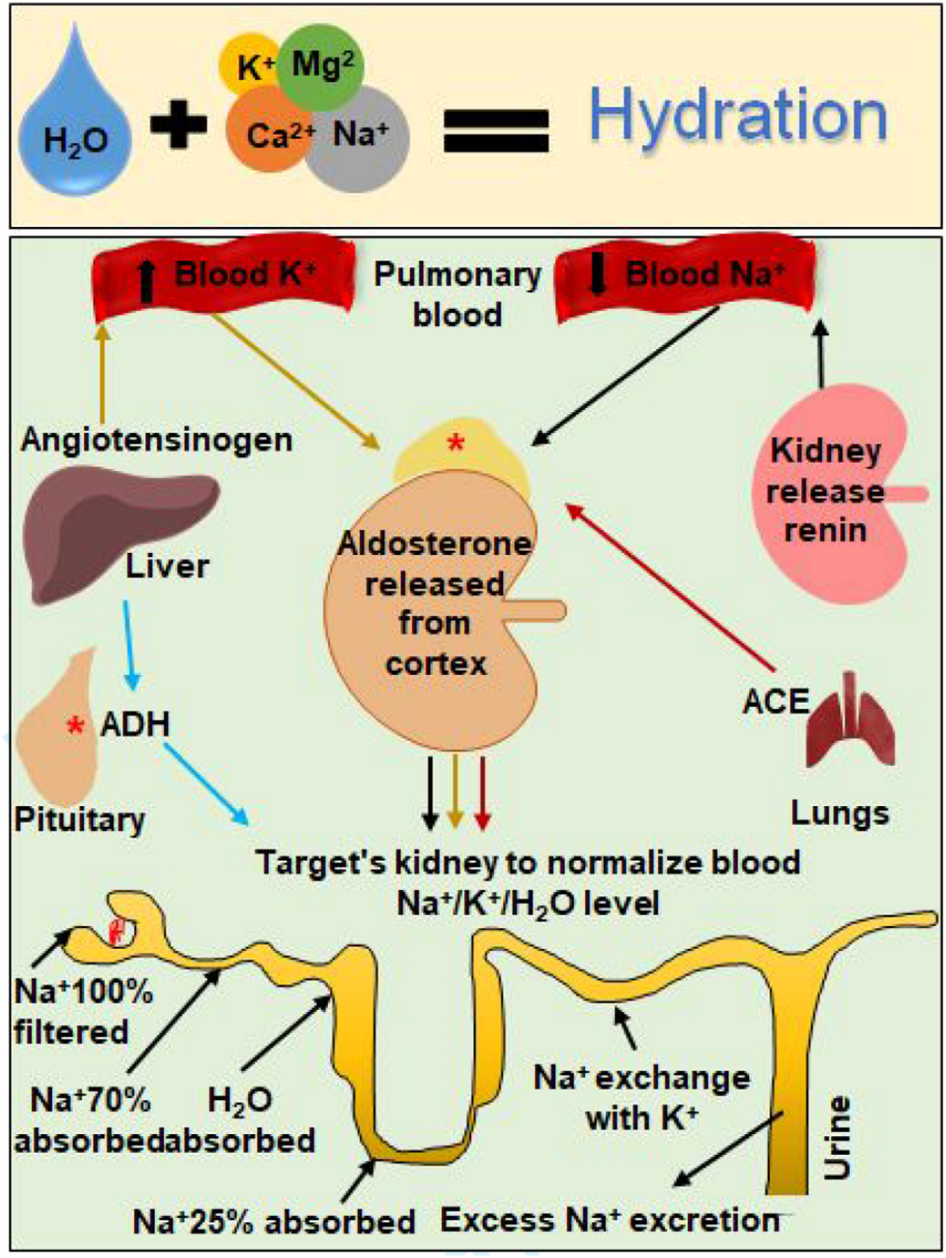
Schematic diagram of electrolytes balance. Electrolytes play a vital role in maintaining homeostasis within the body

Generally, water and electrolytes are associated and are maintained at a homeostatic level by the blood, the lungs, and the kidneys (Figure 2). Mostly, the water and electrolyte abnormalities co‐occur as a result of the primary cause (eg, prolonged vomiting, diarrhea, sweating, or high fever). However, in cancer patients, electrolyte imbalance may occur even in the absence of significant fluid abnormalities due to secondary causes (eg, altering parathyroid hormone [PTH] levels or drug side‐effects). Electrolyte abnormality concentrations in plasma are shown in Table 2. Various factors can induce electrolyte dysregulation^43^ including cancer (eg, brain metastasis, adrenal metastasis, and kidney metastasis), cancer treatment (eg, chemotherapeutic agents, target therapies, and immunotherapies), concomitant drugs (eg, thiazide diuretics, insulin, granulocyte growth factors, beta‐2 agonists, and glucocorticoids), and concomitant diseases (eg, heart failure, kidney failure, thyroiditis, hypercortisolism, liver cirrhosis, pneumonia, and inflammatory lung or brain diseases).

**TABLE 2:** Average electrolyte concentrations in various biofluids

Euhydrated value						
Indices	Plasma	Tear	Urine	Saliva	Sweat	Interstitial fluid
pH	7.3‐7.4	6.5‐7.6	4.5‐8.0	6.2‐7.6	4.5‐7.0	7.35‐7.45
Na^+^ (mM)	135‐145	132	80‐280	3‐29	20‐80	136‐145
K^+^ (mM)	3.5‐5.3	6‐42	24‐79	6.4‐36.6	4‐8	3.5‐5
Ca^2+^ (mM)	4.5‐5.5	0.3‐2	2.3	0.88‐2.5	0‐1	3.4
Mg^2+^ (mM)	0.7‐1.0	0.3‐1.1	1‐5	0.65	0‐2	1.50‐2.5
Cl^−^ (mM)	95‐110	110‐135	91‐150	0‐27	20‐60	110‐118
Osmolality (mOsm/L)	285‐295	302 ± 9.7	500‐850	21‐77	62‐192	280‐296

Imbalance						
Type	Plasma concentration (mM/L)					
Hypernatremia	>145					
Hyponatremia	<136					
Hyperkalemia	>5					
Hypokalemia	<3.5					
Hypercalcemia	>3.5					
Hypocalcemia	<2.1					

**FIGURE 3:**
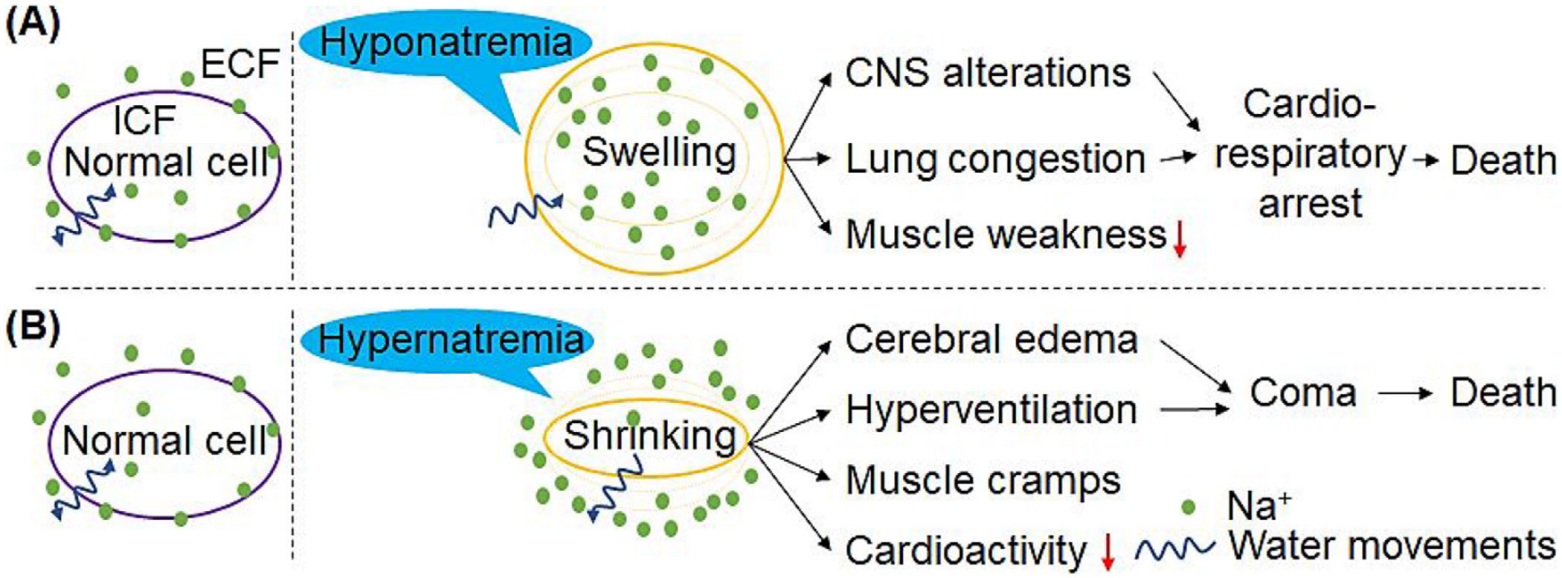
Physiological consequences of (A) hyponatremia and (B) hypernatremia

Hyponatremia was diagnosed in 20 common types of cancer with deleterious effects on brain cells, resulting in neurological symptoms such as headaches, confusion, irritability, seizures, or even coma. In most cancer types, the release of a syndrome of inappropriate antidiuretic hormone (SIADH) is one of the most common causes of electrolyte imbalance, hyponatremia, and hypokalemia frequently reported.^4^, ^44^ Hyponatremia in the small‐cell lung cancer patient is caused by the SIADH, which more frequently developed with other malignancies.^45^, ^46^ This SIADH is driven by the release of arginine vasopressin (AVP, also known an antidiuretic hormone) by cancer or by effects of anticancer medications.^47^ For example, cisplatin enhances AVP secretion to cause SIADH; it directly injures renal tubules and impedes sodium reabsorption, which leads to hyponatremia via renal salt wasting.^48^ Hypernatremia was diagnosed in various types of cancer such as metastatic cancer,^49^ hematological malignancies,^50^ and patients with terminal cancer.^51^ Hypernatremia mainly occurs by two mechanisms in cancer patients: net water loss or excessive salt intake. For example, a recent case study reported that a patient undergoing follow‐up for cervical cancer believed that bay salt would cure cancer. The patient had been taking four teaspoons of bay salt a day, leading to extremely severe hypernatremia.^52^ Figure 3A and B present the physiological consequences of hypo‐ or hypernatremia.^53^, ^54^ Hypernatremia's progress leads to hyperosmolality, forcing the shift of the water from IC to the EC and causing brain cell shrinkage, even vascular rupture, and neurologic deficits.

Hypercalcemia is a common (up to 30%) electrolyte disorder in patients with advanced malignancies and is observed in patients with advanced breast cancer, skeletal metastases, and chemotherapy.^55^, ^56^ Osteolytic hypercalcemia usually occurs due to extensive bone metastases via PTH‐related protein‐mediated mechanisms and commonly occurs in multiple myeloma and metastatic breast cancer. The most common cause of hypercalcemia in various cancers (lung, renal cell, ovarian, thyroid, colorectal, breast cancer, hepatocarcinoma, cholangiocarcinoma, neuroendocrine, and gastrointestinal stromal tumors) are the alterations of PTH levels and release of cytokines (osteoclast stimulating factor) from the tumor.^57^ Those are responsible for osteoclastic activation and increased bone resorption, and also often through increased synthesis of receptor activator of nuclear factor‐κB ligand (RANKL), provoking bone destruction and Ca^2+^ release.^58^ In a recent case study, the patient undergoing four cycles of adjuvant chemotherapy with cisplatin and etoposide for a rhabdoid tumor and showed normal calcium and tumor markers (CA‐125 and NSE). After completing the adjuvant chemotherapy, the patient experienced anorexia, generalized weakness, nausea, and constipation, and in the subsequent examination, malignant hypercalcemia was detected with blood calcium at 5.26 mmol/L.^59^

Cancer medications mainly induce hypokalemia (eg, cisplatin, amphotericin B, and aminoglycoside antibiotics) via tubular injury, which can release significant quantities of intracellular potassium into the extracellular space due to impaired sodium channel function and kidney losses potassium. Hyperkalemia is also induced by chemotherapy in large tumors due to tumor lysis syndrome.^60^ Particularly, patients receiving chemotherapy for hematogenous malignancy can experience acute hyperkalemia due to massive cancer cell death. When cancer cells lyse,^61^ they release potassium, phosphorus, calcium phosphate, and nucleic acids, which are metabolized into hypoxanthine, xanthine, uric acid, and decrease kidney function by renal precipitation of calcium phosphate crystals.^62^, ^63^ The released potassium and calcium accumulate in the body, which leads to hyperkalemia and hypercalcemia and causes serious dysrhythmias. Inappropriate concentrations of electrolytes are signs of a health risk, and for this reason, the measurement of ion levels in physiological systems is an essential criterion in clinical diagnostics.

### 2.3 Possible routes for assessing fluid and electrolyte imbalance

**FIGURE 4:**
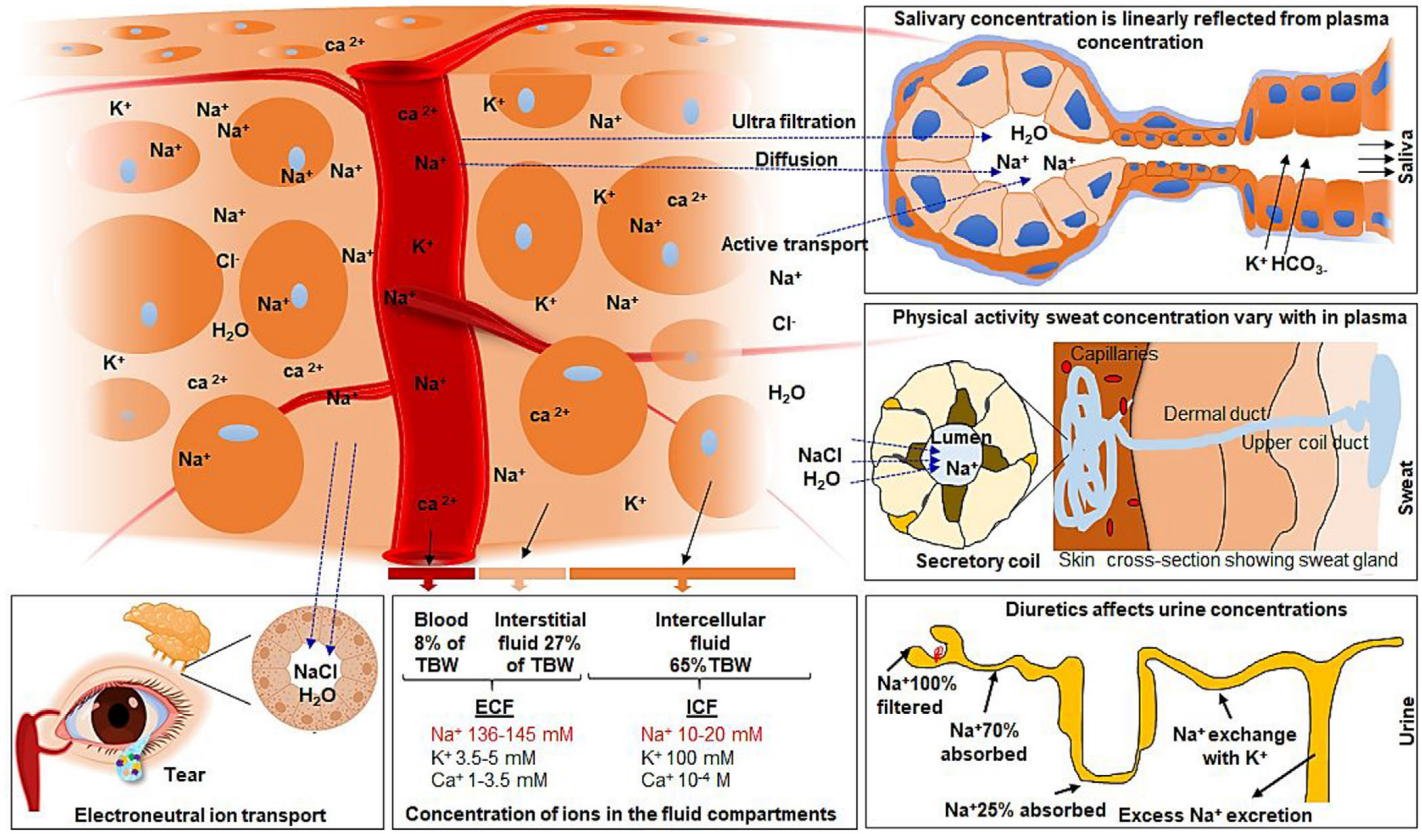
Overview of the fluid and ions that reflect in the bodily system for a possible route for diagnosis

The electrolyte composition of biofluids, such as plasma, tear, urine, sweat, and saliva, is regulated by the metabolic waste of the excretory systems. Changes in Na^+^ concentrations in urine, sweat, tear, and saliva are reflected from ECF concentration^64^ and are depicted in Figure 4. For example, saliva fluid originates from plasma via acinar cells.^65^ The transacinar cell electrolyte (particularly Na^+^) concentration supports the accumulation of primary saliva from plasma through passive and active ion movements across acinar cell membranes (CMs).^65^ During hypernatremia, the ECF Na^+^ concentration increases, reflected in the plasma and might be linked with salivary markers including proteins, metabolites, and electrolytes.^66^, ^67^ In saliva, Na^+^, chloride, and magnesium concentrations are shown to correlate with progressive dehydration.^68^

## 3 DEHYDRATION ASSESSMENT TECHNIQUES

### 3.1 Clinical signs and symptoms assessment

**FIGURE 5:**
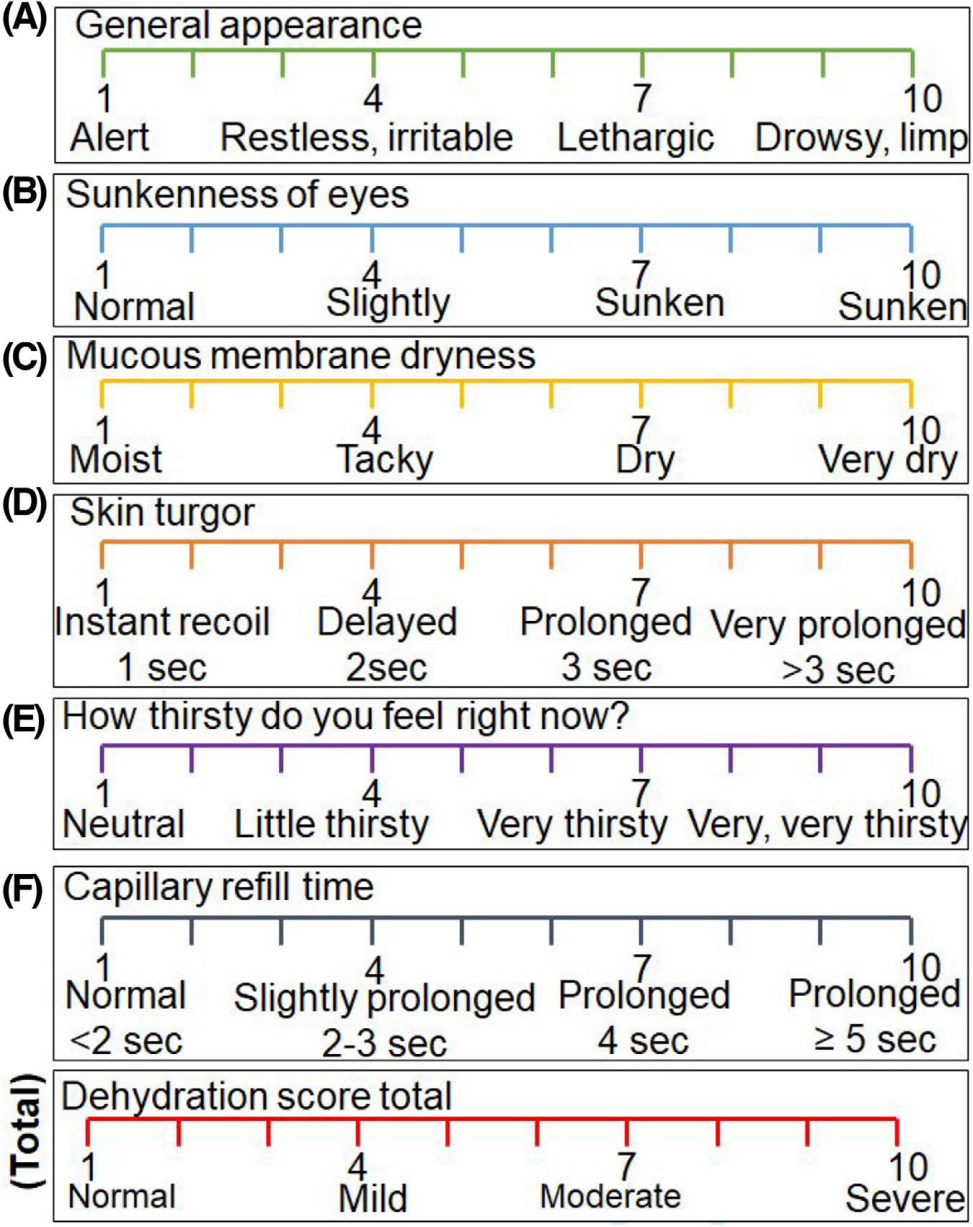
Clinical dehydration scale (10‐point), which is modified from three popular CDS in children with diarrhea.^78^ Scoring: 1: no dehydration, ˂2%; 4: mild dehydration, ˂4% 7: moderate dehydration, ˂7%; 10: severe dehydration, 10%

Signs and symptoms assessment of a patient with fluid imbalance is essential for a correct diagnosis. The assessment techniques that are often cited in the literature for measuring hydration status are the clinical dehydration scale (CDS), and models are provided in Figure 5; the scale range may change. Noncancer studies have found certain variables to correlate with dehydration in elderly patients; these include tongue dryness, upper body muscle weakness, confusion, speech difficulty,^69^ and low body mass index.^70^ The variations in body mass caused by cachexia and edema may make body mass index measurements unsuitable.^71^, ^72^ In a standard medical examination, a capillary refill can only detect hypovolemia in children and lacks sensitivity in adults.^73^, ^74^ The formal CDS consists of four clinical items to predict dehydration. Each scale has its limitations, such as physical examination, which has less sensitivity and specificity^75^ compared to laboratory biochemical testing.

In these instances, the term clinical cancer dehydration is generally used to encompass all types of fluid and electrolyte imbalance as they appear in the clinical setting.^70^ Researchers developed a dehydration score for the cancer population consisting of three variables (eg, mucous membranes dryness, axillary moisture, and sunken eyes).^76^ These signs are shown to correlate with dehydration, as confirmed in elderly patients significantly.^69^ Using the dehydration score, Morita et al. studied the fluid balance and alteration in clinical signs in terminally ill patients with abdominal cancer.^77^ The authors found that BUN/creatinine, sodium, or potassium levels, and fluid balance do not strongly correlate with actual changes in clinical signs of dehydration.^77^ These findings suggest that physical examination inaccurately portrays the dehydration status of cancer patients. Regardless of their limitations, clinicians still rely on signs and symptoms and often diagnose dehydration without considering biochemical findings. The formal measurement methods may need more than four clinical items to predict dehydration categories in clinical populations. To predict dehydration, we are establishing an ideal CDS consisting of six clinical items for more accuracy. The degree of dehydration may be summed for a total score ranging from 0 to 10 (Figure 5). Further research is required to determine the physical signs and symptoms as clinical dehydration indicators of cancer patients.

### 3.2 Osmolarity/osmolality analysis

Osmolality estimates dissolved particle concentration in biofluids, such as plasma, serum, urine, saliva, tear, and sweat, and have been proven to evaluate dehydration. Table 2 provides the human body's euhydrated condition osmolality. Higher osmolality indicates more electrolyte particles in the biofluids, and lower osmolality indicates that they are more diluted. An increased level is associated with Na^+^ load, but they may also develop because of chronic water deficit. The test for plasma osmolality is most commonly used to assist in diagnosing hypernatremia and hyponatremia. Plasma osmolality is the most treasured hematologic parameter to monitor dehydration status and is considered one of the gold‐standard techniques in the clinical setting.^79^ The osmolality of body fluids is usually measured by freezing‐point depression osmometry,^80^ vapor pressure‐based osmometer, cryoscopic osmometer, or calculated from the Equation (1) for plasma and Equation (2) for urine.

(1) $$\begin{array}{ccc} \mathrm{Plasma}\;\mathrm{osmolality} & = & 2\left(\mathrm{Na}\frac{\mathrm{mmol}}{L}+k\frac{\mathrm{mmol}}{L}\right)\\ & & +\;\mathrm{glucose}\frac{\mathrm{mmol}}{L}+\mathrm{urea}\;\mathrm{mmol}/L \end{array}$$

(2) $$\begin{array}{ccc} \mathrm{Urine}\;\mathrm{osmolality} & = & 2\left(\mathrm{Na}\frac{\mathrm{mmol}}{L}+k\;\frac{\mathrm{mmol}}{L}\right)\\ & & +\;\mathrm{urea}\frac{\mathrm{mmol}}{L}. \end{array}$$

**FIGURE 6:**
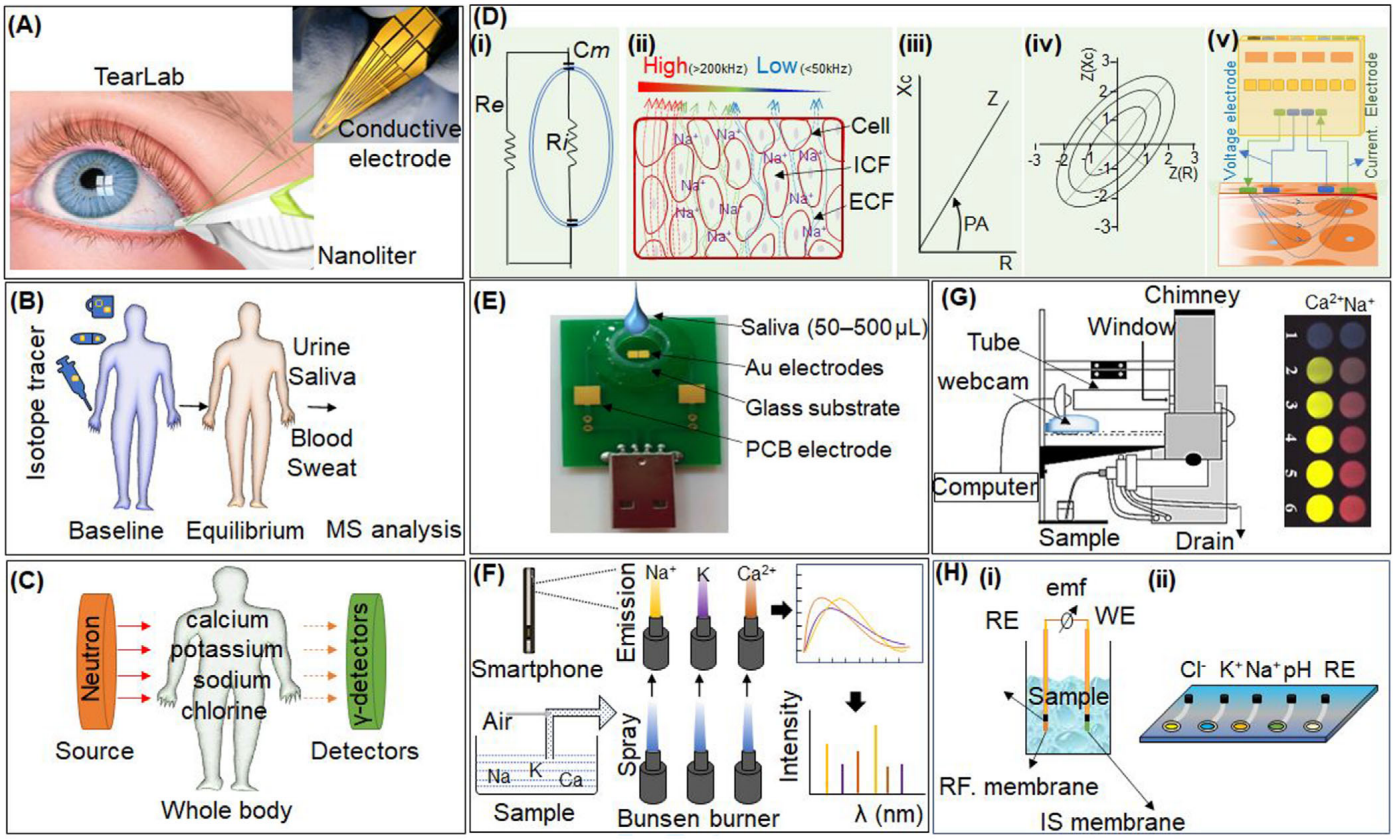
(A) TearLab osmolarity test. Method of obtaining an analysis of tear osmolarity by placing the orifice of the TearLab into the lower lacrimal lake. Images provided with permission from TearLab Corp. (CA, USA). (B) Schematic illustration of in vivo ions/isotope tracer study. One or more stable isotope tracers are introduced into the body. After equilibrium, blood or other body fluid or tissue specimens are collected before and after the tracer infusion. Isotopic enrichment (tracer to trace ratio) of elements of interest is subsequently analyzed by means of GC/MS or LC‐MS. (C) Drawing of the general configuration of in‐vivo neutron activation analysis in clinical diagnosis (modified from Ref. [88]). (D) Bioelectrical impedance analysis. (Gi) Electrical circuit diagram describe the electrical characteristics of the impedance body intra and ECF relationships. (Gii) Current flow at multiple frequencies. (Giii) Graphical representation of bioelectrical impedance analysis raw measurements: Z, Xc, R, and PA. (Giv) Example of a BIVA plotted on the RXc graph with different tolerance ellipses (modified from Ref. [92]). (Gv), Four‐electrode technique using an impedance analyzer. (E) Custom‐made miniaturized portable system to monitor saliva conductivity through Au microelectrode on a glass substrate (Adapted from Ref. [93]). (F) Representation of an atomic emission spectrometer combined with multivariate image analysis. This was capable of on‐site measurements of Na^+^, K^+^, and Ca^2+^ (modified from Ref. [94]). (G) Schematic diagram of a digital image‐based FES method for the quantitative Na^+^ and Ca^2+^ analysis in the serum (adapted from Ref. [95]). (Hi) Schematic of a general potentiometric cell containing a solid‐state ISE and a solid‐state reference electrode, and (ii) a wearable potentiometric ion patch for on‐body electrolyte monitoring (modified from Ref. [96])

Munoz et al. showed the saliva osmolality had been highly correlated with serum osmolality, urine osmolality, urine volume, and urine‐specific gravity.^81^ Therefore, saliva osmolality also can be an effectively diagnosed biomarker during active dehydration. During dehydration, tear osmolality progressively increases, which is reflected in the increase in extracellular tonicity.^20^ However, laboratory‐based measurements require large sample sizes, expensive equipment, and are time consuming.^82^ Tomlinson et al. compare the new OcuSenseTearLab osmometer (Figure 6A, electrical impedance‐based “lab‐on‐a‐chip” nanoliter technology) with the Clifton Osmometer (freezing‐ point depression) to explore the potential of providing clinicians with a readily available clinically applicable measure, which could become the gold standard for fluid biomarker diagnosis.^82^ This instrument utilizes a temperature‐corrected impedance measurement to provide an indirect assessment of osmolarity. The tip of the handheld “pen” is placed in the lower lacrimal lake for 30 s to obtain a reading. The osmolarity is calculated and displayed as a quantitative numerical value, and three consecutive readings are required with the TearLab to obtain a reliable measure of tear osmolarity and the resultes correlates well with the Clifton Osmometer.^82^, ^83^ We would like to point out here those working in dry eye detection using *T*_osm_, tear fluid osmolarity is one of the potential markers of hydration status,^20^ which also proved that *T*_osm_ correlates well with a marker of hydration (plasma osmolality).^84^ So, the TearLab device has the possibility of a non‐invasive dehydration detection tool. Future work towards practical handheld osmometer applications requires detailed validation studies compared to blood and critical assessment of cancer dehydration. Continuous discovery of new handheld osmometers using plasma, serum, urine, saliva, and sweat biomarkers will further help expand the diagnostic scope via osmolality.

### 3.3 Isotope tracer analysis

Stable isotope labeling is a more commonly used technique, and Figure 6B shows a typical schematic representation of a human subject participating in an isotope tracer infusion. The sample with the known concentration of isotopes (usually nonradioactive, deuterium (^2^H), deuterium oxide (^2^H_2_O), or oxygen‐18 (^18^O)); rarely radioactive isotopes, ^22^Na, ^24^Na, ^36^Cl)^85^, ^86^ is administered (generally, one or more stable isotope) either orally or intravenously and allowed to equilibrate for about 3‐4 h. During this time, the isotopes can exchange with hydrogen and oxygen in basal biochemical and metabolic reactions and diffuse the entire body in a matter similar to that observed in water. After this time, the bodily fluid (blood, urine, saliva sample) is taken for analysis by isotope‐ratio mass spectrometry (MS).^87^ Currently, the use of isotope dilution to measure total body fluid (TBF) is limited to the research environment because during daily activities, body fluids are rarely stable, and isotope dilution (ie, deuterium oxide dilution) measurements of TBF require 3 to 5 h for internal isotope equilibration and analysis. Additionally, several factors prevented this technique from becoming a routine clinical method including the inconvenience of blood sampling, the delay (typically several days) in the results of blood analysis by MS, and the cost.

### 3.4 Neutron activation analysis

This activation analysis provides an alternative method of ECF assessment, which can identify and quantify about 70% of all known elements from the samples. A sample is treated with specific radionuclides in a nuclear reactor, which can emit gamma rays during irradiation and measured via radiation detectors. Total body composition is measured by neutron activation (Figure 6C).^88^ This method is mainly established in forensic, geology, archaeology, and biochemistry research. In biofluid, total body chloride, K^+^, and Na^+^ are used to calculate ECV, ICV, and 98% of K^+^ in ICV, and 90% of chloride in ECV are confined in major body compartments,^89^ which is directly proportional to the ICF and ECF volumes. Initially, total body water and six elements (carbon, nitrogen, Ca^2+^, K^+^, Na^+^, and chlorine) were analyzed.^90^ Recently, Liu et al. developed a transportable neutron activation analysis system to quantify elements in vivo with acceptable radiation exposure.^91^ Still, this technology requires proper safety equipment, which is available in limited centers. Continuous discovery of new handheld neutron activation requires more safety analysis clinical use and further expanding of the diagnostic scope towards electrolytes monitoring.

### 3.5 Bioelectrical impedance and conductance analysis

#### 3.5.1 Bioelectrical impedance

Bioelectrical impedance relies on body composition (including physiological and pathophysiological status), and the various applied alternating current (AC) signal frequency response.^97^ Figure 6Di presents an equivalent electrical circuit model that accurately represents the body's impedance characteristics. Depending on the signal frequency current flow through the body (Figure 6Dii), it travels through the electrolytes and water in the ICF and ECF (resistance, R). The stored current gets released at CM, measured as reactance (cellular capacitance, C). Changes in bioelectrical impedance are due to the difference in tissue structure, composition, and health status. Therefore, studying the complex electrical impedance can acquire a lot of information about the physiological and pathophysiological status. ICF (electrically conductive), CM (phospholipid bilayer‐insulator), and ECF (conductive) are made up of different biomaterials that possess various electrical properties, and they provide distinguishable responses to an AC signal. So, the CM behaves as an insulator in an electrically conducting medium. The intracellular and extracellular electrolyte compartment, each with a pure resistor (*R_i_* and *R_e_*, respectively) and CM act as capacitance to the applied AC signal and contribute to capacitive reactance. Thus, the impedance varies from total mass composition, ICF and ECF composition, as well as in physiological and pathophysiological states including inflammation, disease, and cancerous.

BIA has become a popular method for whole‐body composition.^98^ In BIA, phase angle (PA) measurement is the relationship between electric resistance (R) and reactance (Rc), which is directly proportional to the body cell mass. When the body integrity changes, the PA shift significantly. Lower PAs seem to be the steady state with low reactance and CM permeability increases. Geometrical relationship among impedance, reactance, resistance, and PA are shown in Figure 6Diii. The recorded measurements, which reflect the relative contributions of the human body's fluid composition and cellular membranes, are positively associated with reactance and negatively associated with resistance. From the available parameters, body fat mass, hydration status (intracellular, extracellular, and total water content), and electrolyte composition can be measured to determine the overall health status. Following a considerable amount of research on various types of cancer, such as PA shift in breast cancer,^99^ and composition in colon cancer^100^ and lung cancer,^101^ PA changes are believed to be an outcome of overall health status. Age, gender, and body mass are the possible limitations of bioimpedance measurements.

For clinical needs, a whole‐body impedance measurement (bioelectrical impedance vector analysis, BIVA) system has been developed using graphical vectors to provide a visual examination of BIA data. Using this method, impedance (Z) is plotted as a vector from its components R (*x*‐axis) and Xc (*y*‐axis) after being standardized by height (H) (Figure 6Div). BIVA has also been used to study body composition in lung cancer and head and neck cancers.^102^ Recently, Cotogni et al. investigated the impact of parenteral nutrition in individuals with advanced cancer receiving chemotherapy as monitored by BIA with 50 kHz frequency.^92^ The outcome of the reactance, resistance, and PA was significantly associated with survival and significantly improved performance status.

With the advanced development and application of bioelectrical impedance spectroscopy (BIS), various researchers initiated the use of two‐electrode based EIS and four‐electrode based EIS (Figure 6Dv) and multielectrode based EIS with multiple frequency bioimpedance to estimate fluid volumes. Multiple frequency bioimpedance (typically low [5 kHz] and high [>100 kHz]) with multiple regression equations are the advanced application of BIS to predict TBF and ECF in humans.^103^ Earthman et al. used BIS for clinical assessment of the fluid distribution, and found it to be more accurate for measuring changes in fluid volumes.^103^ In recent decades, hydration monitoring by bioelectrical impedance wearable biosensor technology has emerged and launched for health monitoring to track hydration levels in individuals (Table 1). For example, the Healbe GoBe2 wristband collects information using its four sensors (automatic tracking of calorie intake, water balance, and stress) which are in contact with the user's skin and analyzes the data to provide its results on the corresponding mobile application. Such technology can allow users to consistently and independently monitor their hydration status. At the same time, limits of the accuracy have been reported consistently for BIS, for example, underestimated fat‐free mass (for example, hydration) in individuals with cancer who had undergone a significant weight loss^104^ and increased concerns for the value of these predictions in cancer dehydration applications. More research is required to extend this BIS in fluid and electrolyte imbalance in the cancer population.

#### 3.5.2 Electrical conductivity

Fluids are characterized by their ionic strength. If the ion concentrations change in the fluid, the electrical conductivity also changes, an indirect function of osmolarity. Fouke et al.^105^ used a thin‐film microelectronic technology to produce a flexible sensor, which was placed directly onto the ocular surface to calculate the fluids' electrical conductivity. A similar technique was adopted by Ogasawara et al.^106^ who set a conductimetric sensor on the ocular surface, and the tear fluid conductivity was monitored. The sodium chloride concentration of the tears calculated from a calibration curve and converted to the equivalent electrolyte concentration. One obvious benefit of this technique is the in‐situ measurement of osmolarity. The limitation is that the sensor needs to be placed on the ocular surface, reflecting tear secretion, which may alter the tear fluid's osmolarity. Recently, Lu et al.^93^ developed a portable system to monitor saliva conductivity for real‐time dehydration diagnosis, which showed more than 93% sensitivity and 80% specificity (Figure 6E). They indicate that the measured saliva conductivity is consistent with serum osmolality. However, the portable equipment required for continuous monitoring and cancer dehydration measurements is a challenge.

### 3.6 Flame emission spectrophotometry (FES)

In the early days, serum Na^+^ was measured by flame emission spectrophotometry (FES), a diluted serum sample was sprayed on a flame, and the light intensity (specific wavelength corresponding to Na^+^) was measured using a spectrophotometer. Later, the FES was used to detect multiple elements simultaneously with high sensitivity,^107^ which was achieved by a specific spectral signature (discrete emission wavelengths). So, FES is the method of choice for a broad range of applications ranging from trace analysis to a high concentration level of Ca, K, Na, and Mg determination.^108^ The accurate estimation of electrolyte composition in biological samples is of importance in clinical diagnosis. But the main challenges in complex samples, such as urine, is the presence of easily ionized elements, which alter the quantification of low Na^+^ content or trace elements, and show uneven quantification among the samples and need internal standards.^109^ Recently, Debus et al. developed a homemade digital atomic FES platform (Figure 6F) for the determination of Na^+^ content in human urine, which can potentially operate in an on‐site mode.^94^ Additionally, Lyra et al. developed a digital image‐based FES (Figure 6G) for serum chemical analysis including Na^+^ and Ca^2+^. Ions in the air–butane flame emits radiation which is exposed in the acquisition of the digital image by the webcam (detector) on the relation of the Red‐Green‐Blue color pattern.^95^ With this information, plasma/serum Na^+^, K^+^, and Ca^2+^ measurements are sufficient to assess the degree of electrolyte imbalance.

### 3.7 Ion‐selective electrodes (ISE) techniques

Clinically, direct or indirect ion‐selective electrodes (ISEs) methods are commonly used in biochemistry laboratories in order to measure electrolyte concentration.^110^ For direct ISE, the undiluted samples are used, whereas, in the indirect ISE case, the sample is first diluted in a buffer. Of the two techniques, indirect ISE is more widely used. An advantage of diluting the sample is that the measured activity better approximates the concentration, and consequently, indirect ISE results match those obtained using the other biochemistry analysis.^111^ The accuracy of such methods of serum electrolytes estimation has been validated in standard clinical laboratories.^111^, ^112^ However, due to the additional preanalytical ions, the ratio of ion to water in serum is altered erratically, leading to inaccurate evaluations when ions are measured indirectly, which are rare and due to a preanalytical error. Another example is vacutainer tubes containing a Na‐based anticoagulant, such as Na‐heparin, Na‐fluoride, Na‐citrate, or Na‐EDTA, which can markedly elevate plasma Na levels.

Dziewulsk et al.^113^ studied the salivary mineral composition in patients with oral cancer using ISEs (for Na^+^ and K^+^) and colorimetric methods (for Ca^2+^, magnesium, iron, and phosphorus). The highest salivary Na^+^, Ca^2+^, magnesium levels were reported in cases of oral squamous cell carcinoma. The Na^+^ concentrations show statistically significant differences between the study and control groups of cancer populations. This Na^+^ reflection suggested a linear relationship between the saliva and plasma concentrations^114^; the salivary mineral markers would be useful for the diagnosis of electrolyte imbalance in cancer patients in a noninvasive manner because Na^+^, K^+^, and Ca^2+^ are the major cations in unstimulated whole saliva.^115^ For determining the cation and anion concentrations, Abramova et al. developed a solid contact ion sensor with a conducting polymer layer copolymerized with the Ca^2+^ ion‐selective membrane for the determination of Ca^2+^ in serum.^116^ The potentiometric responses from the ion sensor were comparable with optical emission spectrometry results, in which they showed high selectivity and stability of the developed ISEs. A typical solid‐state potentiometric sensor (Figure 6Hi) comprises solid‐state ISE and a solid‐state reference electrode (RE). The measured electromotive force is the difference in electrical potential between the two connectors of the ISE and RE to measure the elements. Recently, wearable potentiometric sensors have received much attention, Parrilla et al. reviewed wearable potentiometric ion sensors for monitoring various biomarkers including Na^+^, K^+^, Ca^2+^, magnesium, ammonium, and chloride.^117^ And they developed a wearable potentiometric ion patch using all‐solid‐state technology for on‐body electrolyte monitoring in sweat. They fabricated an all‐solid‐state flexible electrode array for Cl^–^, K^+^, Na^+^, and pH, together with the RE (proof‐of‐concept, Figure 6Hii).^96^ The results were correlated with the results from gold‐standard techniques. Lim et al. also developed a wireless flexible film‐based system with a Na^+^ sensor using hydrophobic composite, carbon black, and soft elastomer.^118^ The sensor system demonstrates a repeatable analysis of selective Na^+^ detection in saliva, and shows good sensitivity, stability, and selectivity coefficient of Na^+^ against K^+^. Most recently, Sempionatto et al. developed a flexible skin‐mounted microfluidic potentiometric device for simultaneous electrochemical monitoring of Na^+^ and K^+^ in sweat. The device allows efficient natural sweat pumping to the potentiometric detection chamber, containing solid‐contact ion‐selective Na^+^ and K^+^ electrodes. The sweat‐based analysis is still questionable for dehydration in cancer patients because they are mostly physically inactive. However, the traditional ion‐selective method is still used for the assessment of electrolyte concentration in blood in clinical laboratory settings.

### 3.8 Ion‐sensitive fluoroprobes

Multiple probes are commercially available for each ionic species, and some recent approaches described that the ion‐sensitive probes could be used to determine the concentrations of electrolytes in various fluids. Fluorescent probes are the general tool for monitoring ion concentration in living cells, and fluorescence was acquired using various microscopes including whole‐body imaging and intravital microscopy by selecting appropriate emission filters. Typically, Na^+^ concentrations of mammalian cells are 5‐15 mM for cytosol ([Na^+^]_int_) and 120‐150 mM for extracellular ([Na^+^]_ext_) medium. Alterations in intracellular concentrations of Na^+^ and corresponding changes in membrane potential are known to be major actors of cancer progression. Iamshanova et al. developed a methodological study that provided a detailed description of Na^+^ fluorescence imaging in prostate cancer cells and compared the suitability of three Na^+^‐specific fluorescent dyes Na^+^‐binding benzofuran isophthalate (SBFI), CoroNa™ Green, and Asante NaTRIUM Green‐2 (ANG‐2). Of that dye, SBFI and ANG‐2 showed comparable levels of signal intensities after various [Na^+^]_i_ alterations.^119^ Additionally, the cellular Na^+^ level is quantified in human U937 lymphoma cells by flow cytometry using the Na^+^‐sensitive probe ANG‐2), which is currently the most sensitive and popular Na^+^‐sensitive probe.^119^, ^120^

**FIGURE 7:**
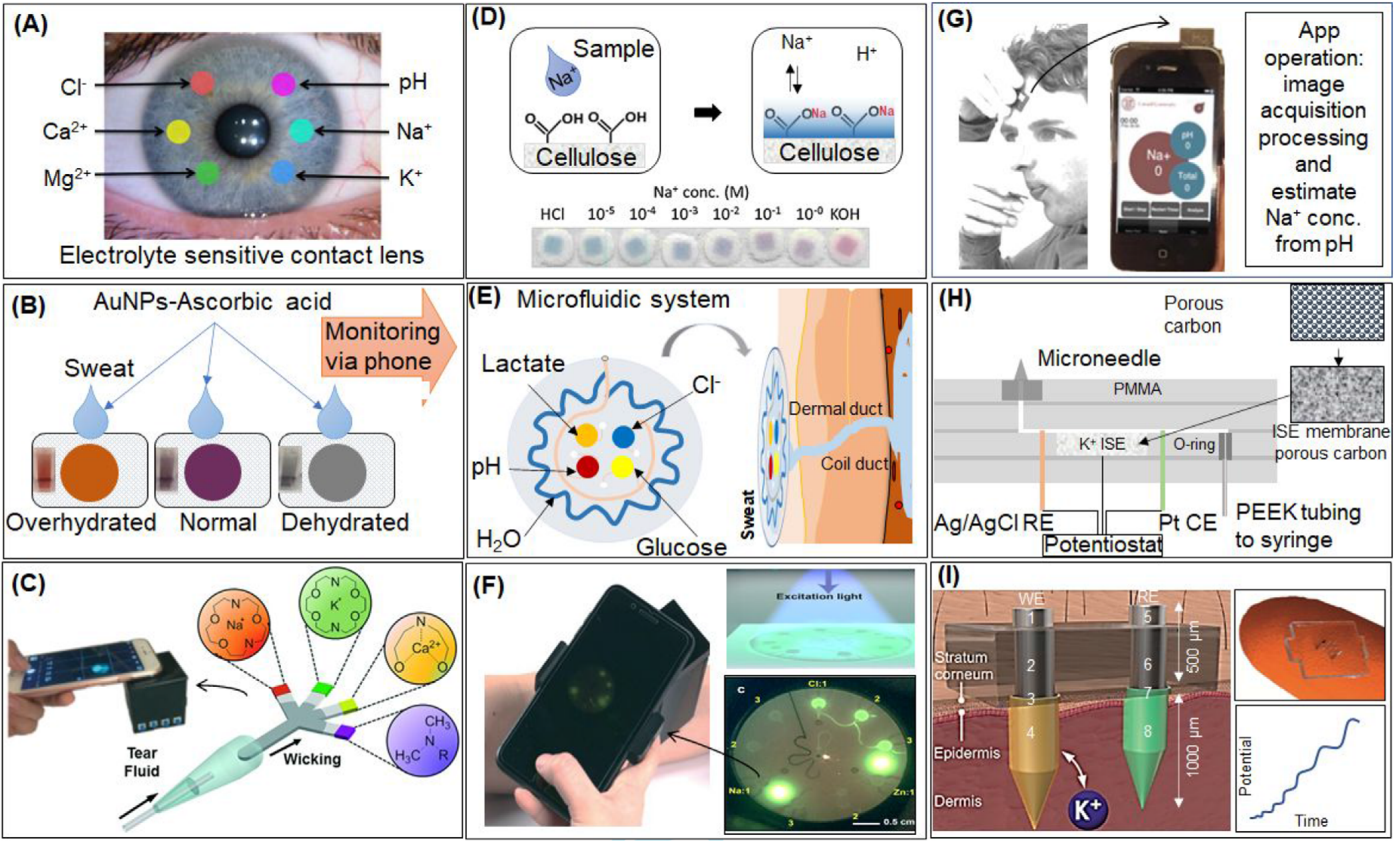
(A) Schematic diagram of contact lens for measurement of a specific single ion by an ion‐sensitive fluorophore (adapted from Ref. [121]). (B) Schematic illustration of the colorimetric sensor, AuNPs solution in the presence of different concentrations of NaCl (modified from Ref. [125]). (C) Cation‐exchange reaction between analyte cations (Na^+^) and protons of carboxyl groups present on the surface of cellulose (adapted from Ref. [127]). (D) Ion‐selective optode system into a paper‐based analytical device for colorimetric Na^+^ detection. Ionophore‐doped plasticized PVC membrane reaches ion‐exchange equilibrium, Na^+^ concentration is confirmed by colorimetric detection (adapted from Ref. [127]). (E) Microfluidic‐based colorimetric chemical analysis represents the sweat‐filled device on skin sweat surface for smartphone‐based signal analysis (adapted from Ref. [128]). (F) Fluorometric skin‐interfaced microfluidic platform for sweat biomarkers analysis. Fluorescent probes selectively react with target biomarkers upon sweat flow through the microfluidic system fluorescence intensity examined by smartphone‐based electrochemical or colorimetric studies (adapted from Ref. [129]). (G) Sweat sample acquired by applying the test strip on the forehead or saliva drooling on the test strip, followed by inserting in the optical system to analyze the pH using the iPhone app (adapted from Ref. [130]). (H) Illustration of a cross‐section of the K^+^ ISE microfluidic chip. The chip with on‐chip reference and counter electrodes with a hollow microneedle which is made by two‐photon lithography (adapted from Ref. [133]). These microneedles provide an opportunity for K^+^ detection in the interstitial fluid at the skin. (I) ISE microneedle for K^+^ detection (adapted from Ref. [22]). Illustration for the working electrode (WE): 1, stainless steel; 2, carbon covering; 3, f‐MWCNTs; 4, K^+^ sensitive layer. Example for the reference electrode (RE): 5, stainless steel; 6, Ag/AgCl; 7, PVB layer; 8, polyurethane

For clinical use, the ion‐sensitive probes technology must provide information about the electrolyte concentrations in a way that is independent of total fluorescence intensity. Badugu et al. developed modern silicone‐hydrogel contact lenses (SiHG) (Figure 7A) to measure individual ion concentrations in tears (for dry eye disease). This study used six polarity‐sensitive fluorophores and showed that the nonpolar and/or amphipathic molecules bind to nonpolar regions of the SiHG lenses.^121^ Such a lens with multiple ion‐sensitive areas could be used to determine concentrations of the major electrolytes in various fluids. We would like to draw those working in dry eye disease detection using electrolyte concentration. Tear fluid osmolarity is one of the potential markers of hydration status,^20^ which also proved that *T*_osm_ increases and correlates well with the marker of dehydration (plasma osmolality).^84^ So, the contact lens with ion‐sensitive fluorophores has the potential to be used as a noninvasive dehydration detection tool. Future work toward practical applications requires detailed validation studies compared to blood and critical assessment of cancer dehydration.

### 3.9 Colorimetric‐based analysis

In the past decade, a vast number of colorimetric‐based analytical devices have been reported^122^ due to their ease of signal visualization, low cost, and user friendliness. Mainly sodium ions are targeted to determine hydration status. The total Na^+^ loss from sweat is a function of whole‐body sweating rate and sweat Na^+^ concentration; thus, Na^+^ loss from sweat and sweating rate can be estimated and expected as a dehydration biomarker.^123^ Recently, Zhou et al. demonstrated a gold nanoparticle‐based colorimetric sensor for rapid detection of dehydration and overhydration through sweat with normal physiological sweat concentration at 40 mM, overhydration at 26.5 mM, and dehydration at 47.9 mM.^124^, ^125^ Sweat that corresponds to overhydration, normal hydration, and dehydration was measured with the different colors of Ascorbic acid capped AuNPs solution (Figure 7B). Recently, smartphone‐ or digital‐based colorimetry was recognized as innovative technology. Yetisen et al. developed a rapid tear analysis system for point‐of‐care settings using metal‐chelating fluorophores to quantify tear electrolytes (Na^+^, K^+^, Ca^++^) (Figure 7C),^126^ and different fluorescence emitted when the various ions in tears reacted with specified fluorophores. The emitted fluorescence intensity at the ion‐free concentration and the ion‐saturated concentration were detected as the reference. In addition to that, Shibata et al. also developed a cation‐selective optode system into a paper‐based analytical device for colorimetric Na^+^ detection (Figure 7D),^127^ which are shown to monitor the Na^+^ in biological samples selectively. The most recent progress wearable device has been exploited using colorimetric signal transduction to monitor target sweat biomarkers. Koh et al. developed a soft, wearable colorimetric microfluidic sweat sampling device for sweat chemical analysis, representing sweat‐filled devices and smartphone‐based signal analysis. The device provides enhanced microfluidic sampling of sweat during exercise with wireless measurement of targets including chloride, pH, and water detection (Figure 7E).^128^ This strategy enables sweat wick from the skin pores from multiple sites at nearly any area of the body. Sekine et al. developed a fluorometric skin‐interfaced microfluidic platform for the measurement of chloride, Na, and zinc in exercise‐induced sweat. Fluorescent probes selectively react with target biomarkers upon sweat flow through the microfluidic system; the fluorescence intensity was analyzed via a smartphone‐based imaging module (Figure 7F).^129^ This technology offers microliter volumes sampling and sensitivity similar to that of laboratory techniques.

Sweat pH can also be correlated with Na^+^ concentration, as demonstrated by the smartphone‐based accessory developed by Oncescu et al., which provides a method for rapid colorimetric detection in sweat and saliva to predict the risk of dehydration during exercise and electrolyte intake.^130^ Figure 7G shows the smartphone‐based accessory for the rapid colorimetric detection of pH in sweat and saliva. The sample is applied on a disposable strip, which consists of the test and reference strip and a flash diffuser. The disposable strip is plugged into the iPhone optical system and analyzed with the corresponding app.^130^ Moreover, this colorimetric sensor can give target information such as the pH and composition analyte. This colorimetric detection takes advantage of its ability to analyze various biofluids. Even though these systems are not meant for the detection of electrolyte imbalance in cancer patients, these works may bridge the technological gap for monitoring electrolyte imbalance in the cancer population.

### 3.10 Intradermal‐based analysis

In this method, mostly electrochemical or ion‐selective sensors are incorporated into a microneedle that provides a minimally invasive approach for creating artificial pores in the skin to reach the ISF in the dermis layer of humans to execute on‐body transdermal detection of electrolytes. In this context, the microneedle‐based electrochemical devices were conceived for K^+^,^22^, ^131^ Na^+^, and pH^132^ detection by potentiometry readout using a polymeric hollow microneedle with a microfluidic chip and a tungsten microneedle. The microneedle sensors are made by reference and working electrodes (WEs), Ag/AgCl thick films, and ZnO thin films on tungsten (W) microneedles, which is a direct label‐free real‐time detection system.^132^ These sensors were successfully applied to the in‐vivo detection. Subsequently, Miller et al.^133^ developed an ion‐selective transdermal microneedle sensor with a microfluidic chip that is capable of collecting ISF by means of hollow microneedles and driving it up to the potentiometric detection of K (Figure 7H) without having direct intradermal sensing. Challenges include complex fluid extraction mechanisms (eg, vacuum suction, microfluidic valves, or liquid prefilling) and require more time for detection because the ISF needs to diffuse to the electrode and stabilize. Most recently, Parrilla et al. developed a wearable microneedle patch for intradermal potentiometric detection of K^+^ in ISF using a K^+^ selective sensor (Figure 7I), which allows for continuous and real‐time intradermal monitoring of K^+^ in individuals.^22^ Stainless steel microneedles supported on a silicon platform were further modified with carbon coating using carbon nanotubes ink and a sensing cocktail for K selective electrode. The analytical performance was characterized in vitro and ex vivo, which demonstrated that the microneedle patch is appropriate for continuous monitoring of K imbalance inside the skin. The drawback of these microneedle patches is their need to be replaced every day.

### 3.11 Metabolome and proteome analysis

**FIGURE 8:**
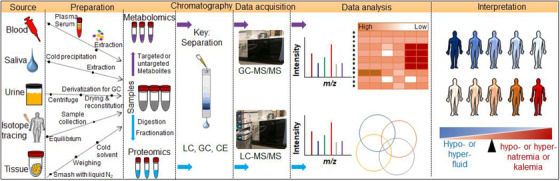
The typical vital steps of a proteomics or metabolomics analysis consist of five stages. Additional steps may be required for specific approaches based on analytical interest. In stage 1, the targeted samples are analyzed and isolated from bodily fluids or tissues by biochemical fractionation or selection. The whole sample analysis is less sensitive than fragmented samples. Therefore, in stage 2, for proteomics, proteins are enzymatically (usually by trypsin) digest to peptides, and for metabolomics, extract the metabolite and derivatization. In stage 3, the samples are separated by one or more steps of chromatography and eluted. After dry, each fraction enters the mass spectrometer, and, in stage 4, a mass spectrum is taken, and the spectra are typically acquired and stored for matching against databases and ensues. In stage 5, data mining involves data preprocessing, interpretation, and compound identification

Figure 8 shows the typical proteomics and metabolomics experiment workflow. In order to study metabolomics and proteomics, MS with electrospray ionization (positive ion mode (ESI^+^) and negative ion mode (ESI^−^)) are widely used to identify and quantify metabolomics and proteomics after optional separation techniques by gas chromatography (GC), high‐performance liquid chromatography (HPLC), or capillary electrophoresis. Most recently, the online nanospray LC‐MS/MS on an Orbitrap Lumos coupled to an EASY‐nLC 1200 system is used for susceptible analyses. Nuclear magnetic resonance (NMR) spectroscopy is also used in modern‐day techniques that do not depend on the sample's separation so that it can be recovered for further analyses. Nontargeted approaches are also widely used. Compared to targeted strategies, nontargeted techniques offer the potential to determine novel biomarkers and seek to detect as many potential features as possible in a single analysis.

#### 3.11.1 Proteomics

Figure 8 shows some key steps which involve proteome analysis, and the steps can be modified in certain approaches based on the techniques or point of interest. The proteomic approaches can be used for proteome profiling and identification of substantial markers of body fluids and electrolytes. Several reports have been published regarding the proteomic and metabolomic characterization of human performance monitoring (ie, exercise‐induced dehydration) from sweat,^134^ saliva,^135^ urine,^136^ and serum.^137^ Most of them are focused on the health benefits to exercise. Despite all benefits, exercise also causes serious illness when it does not follow the clinical or WHO guidance. Exercise can cause homeostatic imbalance, electrolyte disturbances, hydration imbalances, heat‐related illness, and dysnatremia. Several cancers related markers, including ZG16, SLPI were observed in exercise saliva,^135^ associated with electrolyte permeabilities.^138^ ZG16 is decreased in several cancers, such as colorectal cancer or hepatocellular carcinoma,^139^ and further evidence is needed to prove electrolyte imbalance association to cancer. Detrimental effects of exercise on the bodily fluid, NMR based metabolomics,^140^ and proteomic analysis are also useful tools to study electrolyte disturbances. Negatively observed markers were found in saliva samples related to hydration status, amino acids, and other compounds,^140^ which are mostly associated with the electrolyte disturbances.^141^ However, amino acid sequences alone are inadequate in detecting effective biomarkers. A two‐dimensional gel electrophoresis coupled with LCMS/MS has been used to identify novel protein (proteome) biomarkers for exercise mal‐adaptation of whole serum.^137^ The proteomics field has shifted over recent years from traditional approaches to isotopic labeling techniques. For isotope tracer studies, the MS has played a central role by tracking metabolite (ie, amino acids) and protein changes (ie, oxidation or error at the protein N terminus) from stable isotope tracers labeling,^142^ which can also reveal pathway activities for the cause of human cancer.

To study fluid and electrolyte imbalance in cancer, proteome analyses by MS have been employed to identify candidate biomarkers. The marker associated with fluid and electrolytes, such as aldosterone (associated with Na^+^ and K^+^ regulate)^143^ and dehydrogenase states,^29^ are identified. However, very limited studies have been explored to quantify protein expression in cancer dehydration (expression profiling of ion channel genes).^144^ For example, voltage‐gated potassium (K^+^) (Kv) channels and calcium (Ca^2+^)‐activated K^+^ (K_Ca_) channels are known to control cancer cell proliferation via the modulation of membrane potential in breast, colon, and prostate cancers. However, ion channels' overall impact and electrolyte imbalance diagnosis on tumorigenicity in cancer remain to be defined through proteomics.

#### 3.11.2 Metabolomics

Figure 8 shows some key steps which involve metabolome analysis, and the steps can be modified in certain approaches based on the techniques or point of interest. The sample preparation and data analysis are equally important for metabolome analysis. Successful metabolomics starts with picking the right experiment. Menni et al. performed serum metabolomic profiling to identify potential biomarkers and pathways associated with serum electrolyte levels. Metabolite associations with hypo‐ or hypernatremia or kalemia or chloremia are identified as novel metabolites marker, which is significantly involved and distributed across amino acids, cofactors & vitamins, carbohydrates, lipids, nucleotides, peptides, xenobiotics.^141^ From the amino acid groups, the expression of N‐acetyl‐amino acid has been identified in prostate cancer,^145^ which has been associated with hypernatremia.^141^ Additionally, plasma N‐acetylputrescine is recognized as a potential biomarker of lung cancer,^145^ which is also associated with hyponatremia.^141^

Furthermore, there are reports of specific biomarkers profiles, such as IL‐8 and E‐selectin (fluid overload) and intercellular adhesion molecules (salt distribution), that may play a significant and discriminatory role for a better understanding of fluid imbalance and other pathophysiological responses.^146^ Some studies suggest salivary biomarkers, including metabolome, which could provide biochemical and genetic profiling of hydration status. The scope of new noninvasive metabolome profiles can be expanded to additional metabolites and key electrolytes whose concentrations in saliva, tear, and urine display close relationships with those in blood. For example, a direct saliva‐based noninvasive biofluid method of capturing the oncometabolite may improve the diagnosis of cancer‐associated dehydration, since saliva carries a broad spectrum of proteins/peptides, nucleic acids, electrolytes, and hormones that originate from multiple local and systemic sources. Biofluids contain thousands to tens of thousands of metabolites signatures, the most abundant of which are amino‐acid metabolites, pro‐hydroxy‐pro (urea cycle pathway), tricarboxylic acid cycle intermediates, and metabolites (lipid metabolism pathways), which independently associate with Na^+^, chloride, K^+^, and bicarbonate.^141^ Saliva samples could also be used to detect electrolyte biomarkers for cancer via metabolomics.

## 4 CONCLUSION AND FUTURE PROSPECTIVE

Current hydration monitoring for individuals with cancer is difficult because thirst and physical signs of dehydration are often absent or misleading. Such challenges lead to unplanned emergency room or clinic visits and may cause caregivers to question whether clinically assisted hydration is required or not. On the other hand, individuals with cancer are at high risk for dehydration from both cancer and its treatments, and hydration at the end of life (actively dying phase) is not indicated in most cases.^147^ Thus, fluid and electrolyte balance is critical during cancer therapy for enhanced quality of life, influence on actual symptoms and symptom management, and determination of potential treatment options. The most significant laboratory abnormality is Na^+^ imbalance, which should be carefully monitored, and should be normalized very slowly with rehydration therapy.^148^ Currently, no reliable techniques are available to measure fluid and electrolyte imbalance based on pathophysiological status. Of the various available indices, electrical bioimpedance, saliva, tear, and urine are noninvasive and easily accessible for the evaluation of dehydration status and the screening of biomarkers that may reflect the body's overall health status. The identification of dehydration biomarkers is a promising tool to explore in exercise health and disease research, especially in noninvasive samples. Some studies also suggest that salivary proteomic and metabolomic approaches could provide both potential disease mechanisms and disease (fluid and electrolyte abnormalities) biomarkers. The proteomic and metabolomic alterations in response to fluid and electrolyte imbalance are poorly explored; thus, if well researched, targeting noninvasive biomarkers may further contribute to the field of exercise physiology, oncology, and diverse etiology to assess hydration. Combining proteomics analysis with metabolic profiling is especially attractive since metabolomics provides information informing a functional interpretation of proteomics data, while proteomics analysis contributes to a better understanding of metabolomics data by highlighting participating enzymes and or enzymatic pathways. The combination of proteomics and metabolomics could elucidate various molecular mechanisms including dehydration in the cancer population. However, no combined “omics” studies of the electrolyte alterations associated with cancer have yet been performed.

Thus far, research has generated significant progress in molecular and physical technologies for monitoring fluid and electrolyte imbalance. Particularly, monitoring device potentialities, biomarkers discovery, and skin‐integration techniques can provide clinically relevant information in various biofluids such as sweat, tears, saliva, ISF, and urine diagnostics. Advanced research focused on developing multiplexed microfluidic devices, miniaturized electrochemical biosensors, calorimetric devices, bioimpedance, skin biosensors, proteomics and metabolomics profiling will improve the ease of monitoring. None of these modern technologies will be viable without clinical validation and testing. From a research perspective, consumer's (de)hydration tracking technologies can be categorized into those used in discovering biomarkers, validation studies, observational studies, monitoring of health performance, and intervention studies. For effective monitoring of health performance and to observe actual changes in health outcomes, it is essential to specify the study populations with respect to the intended use of the technologies; it is also critical that consumer monitoring technologies provide valid, accurate, and reliable data. Suppose the technologies have been designed to monitor particular health conditions (eg, natremia). In that case, it is important for studies to include individuals from the natremia population (including hyponatremia and hypernatremia) and healthy individuals for comparison so that scientific validation may be more achievable to monitor individual health conditions. Given the competitive research, we anticipate exciting new developments to soon find proper biomarkers of whole‐body electrolyte status during dehydration in humans, including the cancer population, which could prevent (de)hydration, decrease healthcare expenditures, improve patient care and quality of life. Looking to the future, the fast‐growing technological advances that enable the development of health and cancer (de)hydration continuous monitoring systems could provide a way to monitor hydration from the convenience of the patient's home.

## CONFLICT OF INTEREST

The authors declare no potential conflict of interest.

## AUTHORS CONTRIBUTIONS

FZ: Conception, design, review & editing the manuscript, funding acquisition, resources, project administration, and supervision; DB: Conception, design and drafting, review & editing the manuscript; YK, JP and AM: review & editing; PP: Made important suggestions, review & editing. All authors discussed, reviewed, and approved the final manuscript.

## Data Availability

Data sharing is not applicable to this article as no new data were created or analyzed in this study.
